# Dominant cytotoxic NK cell subset within CLPD-NK patients identifies a more aggressive NK cell proliferation

**DOI:** 10.1038/s41408-018-0088-1

**Published:** 2018-06-05

**Authors:** Gregorio Barilà, Antonella Teramo, Giulia Calabretto, Chiara Ercolin, Elisa Boscaro, Valentina Trimarco, Samuela Carraro, Matteo Leoncin, Cristina Vicenzetto, Anna Cabrelle, Monica Facco, Francesco Piazza, Gianpietro Semenzato, Renato Zambello

**Affiliations:** 1 Department of Medicine, Hematology and Clinical Immunology Section, Padua University School of Medicine, Padua, Italy; 2 Venetian Institute of Molecular Medicine (VIMM), Padua, Italy

Natural killer (NK) cells represent a class of innate lymphocytes with large granular morphology and cytotoxic functions, characterized by the CD3–/CD16+/CD56+ phenotype. According to CD56 expression, two major NK cell subsets can be recognized, CD56^high^/CD16^dim/neg^ NK cells with the ability to release cytokines and CD56^dim^/CD16^high^ NK cells displaying cytotoxic ability toward virus infected or neoplastic cells^1^. NK cells are traditionally considered part of innate immunity but evidence has been recently provided that a distinct NK cell subset may respond to specific antigens like adaptive immune cells^2^. These NK cells, also known as “NK memory”, are induced by the chronic stimulation of viral infections or by cytokines (IL-12, IL-15 and IL-18) and are included in the CD56^dim^/CD16^high^ NK cells subgroup equipped with CD57 but lacking CD62L^2,3^. Chronic lymphoproliferative disorder of NK cells (CLPD-NK) is a provisional entity, recognized by the 2016 WHO classification, characterized by chronic expansion of at least 500/mm^3^ NK cells with restricted killer immunoglobulin-like receptor (KIR) pattern, whose assessment is of crucial relevance due to the lack of T-cell receptor rearrangement in NK cells^4,5^. CLPD-NK has generally an indolent course, most patients being asymptomatic and the main feature of the disease being represented by the development of neutropenia^4^. Only few data are available on the pathogenesis of this indolent disorder but a constitutive activation of anti-apoptotic signaling pathways is likely to play a relevant role^5^. The discovery in 2012 of somatic *STAT3* mutations, in about 40% of T-cell large granular lymphocyte leukemia (T-LGLL) and in about 30% of CLPD-NK, focused the attention on a constitutive activation of Janus Kinase-Signal Transducers and Activators of Transcription (JAK-STAT) pathway in the development of this disorder^6,7^. In addition, somatic *STAT5b* mutations were recently recognized in rare aggressive variants of LGLL and indolent CD4+ T-LGLL^8^.

This rare disorder remains an extremely heterogeneous disease, some patients being completely asymptomatic, whereas others require specific treatment. For this reason, using flow cytometry and regardless of KIR expression, the aim of this study was to identify different biological and clinical CLPD-NK patients’ subsets. By flow analysis, NK cells of 25 patients affected by CLPD-NK were analyzed for CD16 and CD56 expression (Supplementary Methods), recognizing two major NK cell subsets, that is, patients with CD56^dim^/CD16^dim^ NK cells (4/25, 16%) and patients with CD56^neg/dim^/CD16^high^ NK cells (21/25, 84%) (Figs. 1a–c). All patients were evaluated for clinical and hematological characteristics. Median age was 62 years (range 42–79) with no significant difference between CD56^dim^/CD16^dim^ and CD56^neg/dim^/CD16^high^ subgroups. As expected, neutropenia (absolute neutrophil count (ANC) < 1500/mm^3^) was the most relevant feature, detected in 10 out of 25 patients (40%), with 5 patients (20%) presenting severe neutropenia (ANC < 500/mm^3^). Anemia and thrombocytopenia have been detected only in a minority of patients (2/25 and 3/25, respectively) and were generally mild (Table 1). In terms of clinical presentation, almost all symptomatic patients were included in the CD56^neg/dim^/CD16^high^ subgroup with 8 out of 21 (38%) patients presenting neutropenia and 5 out 21 (24%) severe neutropenia. Only three patients required treatment (low-dose cyclophosphamide in two cases and methotrexate in one) during the natural history of the disease, all belonging to CD56^neg/dim^/CD16^high^ subset.

**Fig. 1:**
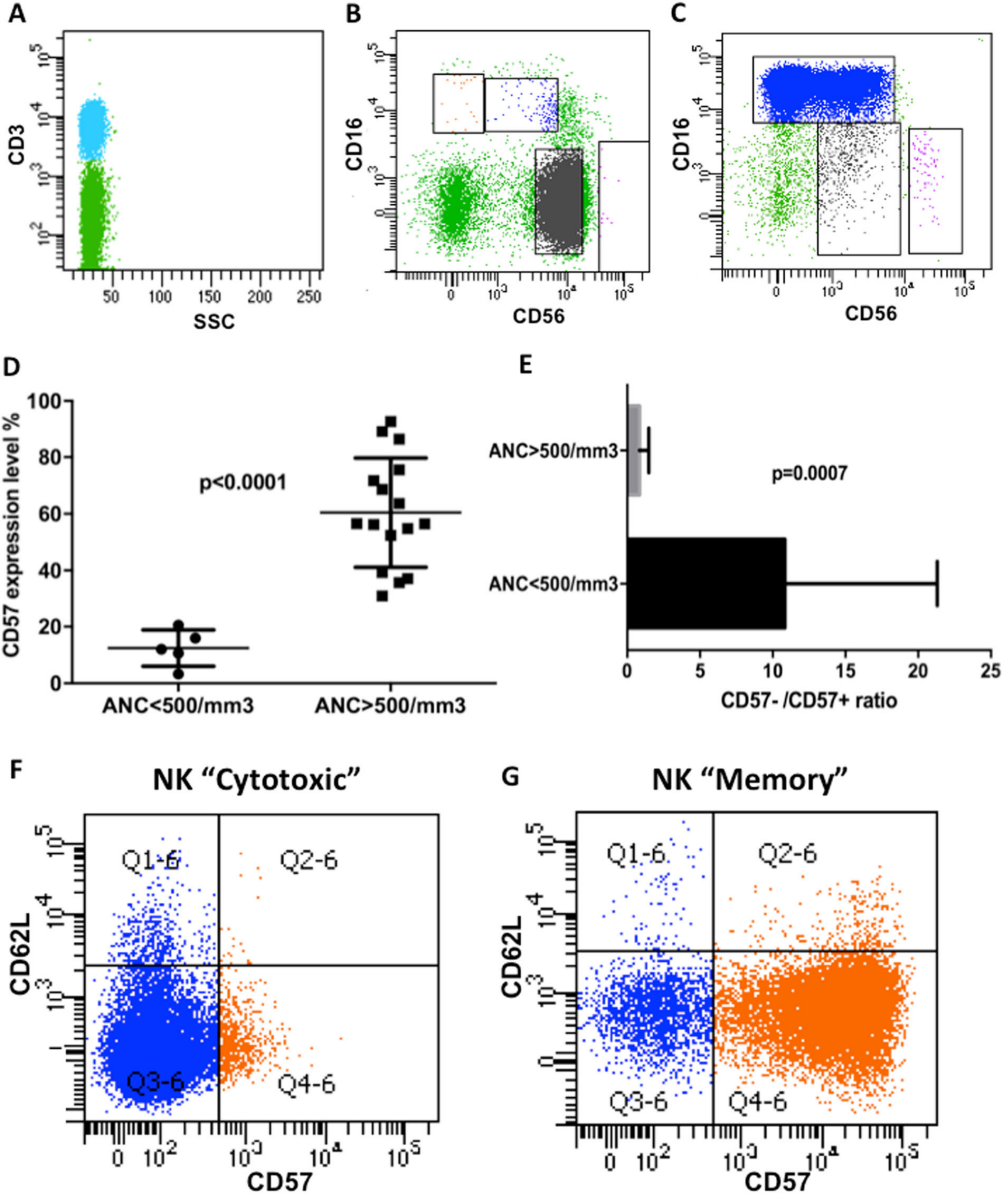
Immunophenotypic features of CLPD-NK patients and their correlation with neutrophil count. **a–c** Immunophenotypic analysis of NK cells. CD3+ lymphocytes were excluded from the analysis (panel **a**, green). CD3- lymphocytes (panel **a**, blue) were studied for CD56 and CD16 relative expression identifying CD56^high^/CD16^dim/neg^ NK cells (pink), CD56^dim^/CD16^dim^ NK cells (gray) and CD56^dim/neg^/CD16^high^ NK cells (blue). As a consequence, patients were classified according to the predominant NK cell subset, of notice CD56^dim^/CD16^dim^ NK cell subgroup (panel **b**) and CD56^dim/neg^/CD16^high^ NK cell subgroup (panel **c**). **d, e** Dot plot indicating differential CD57 expression (%) in CD56^dim/neg^/CD16^high^ subgroup between patients who experienced severe neutropenia (ANC < 500/mm^3^) and patients who did not (ANC > 500/mm^3^). As represented, patients with ANC < 500/mm^3^ presented CD57% expression significantly lower (*p* < 0.0001) toward patients with ANC > 500/mm^3^ (panel **d**). As a consequence, patients who experienced severe neutropenia presented a CD57–/CD57+ mean ratio significantly higher towards patients who did not (*p* = 0.0007; panel **e**). **f, g** Immunophenotypic analysis for CD57 and CD62L among CD56^dim/neg^/CD16^high^ NK cells (blue). CD57 expression (orange) discriminates two patient’s subgroups characterized by CD57 negativity (panel **f**) and positivity (panel **g**) respectively, which we identify as “Cytotoxic” and “Memory” NK subgroups. As previously demonstrated, no significant CD62L expression was found, confirming that both subsets are constituted by terminal differentiated NK cells

**Table 1:** Clinical features of CLPD-NK patients

	**CD56**^**dim**^**/CD16**^**dim**^ **(*n*** **=** **4)**	**CD56**^**dim/neg**^**/CD16**^**high**^**/CD57+ NK “Memory” (*n*** **=** **16)**	**CD56**^**dim/neg**^**/CD16**^**high**^**/CD57- NK “Cytotoxic” (*n*** **=** **5)**
CD94/NKG2A	4/4 (100%)	13/16 (81%)	5/5 (100%)
CD94/NKG2C	1/4 (25%)	4/16 (25%)	0/5
CD158a	1/4 (25%)	0/16	0/5
CD158b	0/4	8/16 (50%)	0/5
CD158e	0/4	3/16 (19%)	0/5
Neutropenia	2/4 (50%)	3/16 (19%)	5/5 (100%)
Severe neutropenia	0/4	0/16	5/5 (100%)
Treatment	0/4	0/16	3/5 (60%)
Anemia	0/4	0/16	2/5 (40%)
Thrombocytopenia	0/4	2/16 (13%)	1/5 (20%)
*STAT3* mutation	0/4	0/16	2/5 (40%)
*STAT5b* mutation	0/4	0/16	0/5

Considering the clinical heterogeneity of CD56^neg/dim^/CD16^high^ subset, we analyzed the expression of CD57 on cells of these patients identifying a 51 ± 13.5% mean expression positivity. Of notice, all five patients who experienced severe neutropenia and all symptomatic patients requiring treatment presented a significantly lower CD57 mean expression toward other CD56^neg/dim^/CD16^high^ patients (12.48% ± 2.87 *vs* 60.43% ± 4.38, *p* < 0.0001, Fig. 1d). As a consequence CD57–/CD57+ ratio was significantly higher in symptomatic patients as compared with remnant CD56^neg/dim^/CD16^high^ patients (10.84 ± 5.24 *vs* 0.84 ± 0.33, *p* = 0.0007, Fig. 1e). Taken these data together, through immunophenotype, three major NK cell subgroups of patients can be identified. CD16 levels discriminated between CD56^dim^/CD16^dim^ subgroup and CD56^neg/dim^/CD16^high^ subgroup. Among the latest, CD57 expression identified patients who experienced severe neutropenia characterized by a “Cytotoxic” phenotype with CD57 negativity from less symptomatic patients characterized by CD57 expression, resembling the “NK Memory” phenotype (Figs. 1f, g, respectively). All the three subgroups displayed high CD94 expression, whereas only CD56^dim^/CD16^dim^ NK cells expressed discrete amount of CD62L. No statistically significant differences in CD94-NKG2A/C and KIR expression were found among the three subgroups although CD94/NKG2C phenotype and KIR restriction for CD158b and CD158e were almost exclusively distinct features of NK “Memory” subgroup (8/16 and 3/16, respectively), whereas CD56^dim^/CD16^dim^ and NK “Cytotoxic” subgroups displayed CD94-NKG2A phenotype and presented a skewed KIR pattern characterized by lack of KIR expression (3/4 and 5/5, respectively). All these features are reported in Table 1.

*STAT3* exon 21 mutations analysis and *STAT5b* exons 16 to 18 analysis were performed in our cohort of patients (Supplementary Methods). As previously reported^9^, the frequency of *STAT3* mutated patients was lower with respect to T-LGLL with only two (8%) mutated patients found. These latters were characterized by the “Cytotoxic” NK immunophenotypic signature without dominant KIR expression and NKG2A pattern. In addition, all mutated patients presented severe neutropenia and required treatment during the natural history of the disease. None *STAT5b* mutated patient was found in our cohort of CLPD-NK patients (Table 1).

With the aim of identifying biological features matching with clinical characteristics, we focused on the recognition of a specific NK cell immunophenotypic signature that eventually allows a biological and clinical classification of this rare disorder. We found that, independently from KIR expression, patients characterized by CD56^neg/dim^/CD16^high^/CD57- cytotoxic NK cell expansion represent a different phenotypic subgroup characterized by symptomatic disease and by the presence of *STAT3* mutation, suggesting a more aggressive NK cell proliferation. These findings agree with the article of *Morice* et al.^10^ in which CLPD-NK patients were classified upon CD56 expression; CD56^neg^ NK cell patients were characterized by CD16 expression, presence of cytopenia and treatment requirement. However, this analysis alone may not be enough considering that also among CD56^neg/dim^/CD16^high^ patients, a different clinical behavior can be recognized. We found that patients who experience severe neutropenia and eventually require treatment belong to the CD56^neg/dim^/CD16^high^/CD57– NK subset, thus conferring a prognostic role to CD57. As a further confirmation, the specific CD56^neg/dim^/CD16^high^/CD57– immunophenotypic signature is also partially associated to a specific biological hallmark of LGL leukemia, which is the presence of *STAT3* mutation. As we recently demonstrated in T-LGLL^11^, also in this large cohort of CLPD-NK patients, immunophenotypical analysis can identify patients with symptomatic disease and might represent a suitable surrogate of *STAT3* mutation sequencing. WHO 2016 classification confirmed CPLD-NK as a provisional entity, emphasizing the high heterogeneity of the disease^4,7,12^. The frequency of *STAT3* mutated patients found in our cohort is slightly lower as compared with what has been reported in previous studies by Jerez and Rajala^7,12^. This feature can be explained by a higher frequency of symptomatic and treated patients in their study populations, possibly due to enrichment in NK “Cytotoxic” patients, as herein defined. Unfortunately, in the above articles^7,12^, the frequency of CD57 NK cells was not reported.

Clonality assessment for NK cell neoplasms still represents an Achilles heel due to the lack of a clonotypic structure on these cells. Besides, only a small fraction of CLPD-NK reveals *STAT3* mutation, the majority of patients displaying wild-type *STAT3*. For this reason, other biological features were assessed to prove the clonal proliferation. Historically, a skewed pattern of KIR expression was considered as a reliable surrogate of clonality to characterize pathological NK cell expansion^13,14^. On the contrary, Bàrcena and colleagues^9^, using HUMARA assay to distinguish monoclonal *vs* polyclonal proliferation, recognized a surrogate for NK clonality in CD94^high^/HLA-DR+ signature. This approach was able to identify a concordance between the presence of *STAT3* mutation and monoclonal NK cell proliferation but, surprisingly, not significant differences were found between monoclonal and polyclonal patients in terms of clinical features (specifically the presence of cytopenia), suggesting that a percentage of truly CLPD-NK symptomatic patients were missed by this classification. In addition, CD94 expression represents a specific marker of mature NK cells that is not suitable for distinction between polyclonal and monoclonal NK cell proliferations.

The etiology of LGLL is still unknown but several authors hypothesized that chronic antigenic stimulation can trigger an initial LGL proliferation that subsequently carries on as a consequence of persistent cytokine stimulation or gain of somatic mutation (i.e., *STAT3* mutation)^5^. The evidence that some patients are characterized by NK cells expansion with memory-like phenotype, may support this hypothesis. In fact, several studies highlighted that viral stimulation by cytomegalovirus can trigger a CD57+/NKG2C NK cell expansion with memory properties and, in some cases, this process is stimulated by cytokines, like IL-12, IL-15 and IL-18, which have been proven to play a pathogenetic role in LGLL^15^.

In conclusion, through NK cell subsets flow analysis, discrete subtypes of CLPD-NK can be identified. At variance with KIR expression, which does not correlate with clinical features, patients characterized by CD56^neg/dim^/CD16^high^/CD57– cytotoxic NK cells expansion represent a unique phenotypic subgroup characterized by more symptomatic disease and the presence of *STAT3* mutation, suggesting a more aggressive proliferation of NK cells.

## Electronic supplementary material

Supplementary Material
