# Supplementary material for: Dominant cytotoxic NK cell subset within CLPD-NK patients identifies a more aggressive NK cell proliferation
Source: Blood Cancer J. 2018 Jun 5;8(6):51. doi: 10.1038/s41408-018-0088-1 (PMC6002482; doi:10.1038/s41408-018-0088-1)
Supplement: Supplementary file 1 — Supplementary Material [file 41408_2018_88_MOESM1_ESM.doc]

**SUPPLEMENTARY**

**METHODS**

**Study patients**

This study followed the Declaration of Helsinki and all patients gave written informed consent. Approval for evaluation and publication was obtained from Padua Ethical Committee board. The study population accounted for 25 patients affected by CLPD-NK from Hematology Unit of Padua. All these patients met the 2016 WHO (World Health Organization) criteria for CLPD-NK diagnosis with NK LGL KIR-restricted expansion persisting for at least 6 months.

**Flow cytometry analysis**

The frequency of LGLs positive for the characteristic antigens was assessed by flow cytometry analysis using direct or indirect immunofluorescence assay combining 6 fluorescences. Briefly, cells were stained with the appropriate mAbs, scored using a FACS Canto analyzer (BD Biosciences, San Jose CA) and data processed by the BD FACS Diva software program (BD Biosciences). The investigation for NK cells surface markers was performed on separated PBMCs. NK cells were stained with fluorochrome conjugated antibodies for CD3 (APC CyTM7), CD16 (PE-CyTM7), CD56 (APC), CD57 (FITC) (BD Biosciences) and CD94 (PE) and CD62L (PE-CyTM5) (BD Pharmingen). CD3+ T lymphocytes were excluded from the analysis. Remnant CD3- lymphocytes were studied for CD16, CD56, CD57, CD62L and CD94 relative expression. Cells with midpoint fluorescence expression below the fourth decade were considered with “high” antigen expression while cells with midpoint fluorescence expression under the third decade were considered with “dim” antigen expression.

KIR and CD94-NKG2A/NKG2C expression was studied using CD158a (FITC), CD158e (PE) (BD Biosciences), CD158b (PE), CD94 (FITC) (BD Pharmingen), NKG2A (PE) and NKG2C (APC) (R&D Systems) monoclonal antibody.

**Isolation of LGLs from patients with CLPD-NK**

PBMCs were obtained by Ficoll-Hypaque (Sigma Aldrich) gradient centrifugation. Patients’ LGLs were obtained using magnetic separations over columns (MACS; Miltenyi Biotec, Auburn, CA) with magnetic Micro-Beads coated with monoclonal anti-human CD57, CD56 or CD16 antibodies (Miltenyi Biotec). Alternatively, LGLs were obtained from PBMCs by the FACSAria cell sorter (BD Biosciences) making the selection according to CD57, CD56 or CD16 antigen expression. Sorted populations were analyzed for purity and viability (both > 95%). In previous articles we ruled out that cells obtained by FACSAria sorting or by magnetic MicroBeads were functionally different^11^.

**Screening for *STAT3* and *STAT5b* mutations**

For the screening of *STAT3* and *STAT5b* mutations we used the set of primers reported by Koskela et al^6^ and by Rajala et al^7^, respectively, to amplify the hot spot regions for mutations (exon 21 for *STAT3* and exons 16-18 for *STAT5b*). DNA from purified LGLs and from the remaining autologous PBMCs was separately analyzed. Genomic DNA was extracted from 1-20 x 10^6^ PBMCs using the Gentra Puregene Cell Kit Plus (Qiagen; Hilden, Germany). DNA was sequenced using dye terminator technology and an ABI 3130 sequencer (Applied Biosystems; Foster City, CA). Sequences were analyzed using Chromas Pro and Blast search. The presence of D661Y and Y640F mutations undetectable by direct sequencing, because of the limited sensitivity of the method (reaching 25% of positive cells, as previously established^11)^, was also analyzed by a DNA tetraprimer amplification refractory mutation system assay (ARMS-PCR), as reported by Jerez et al^7^.

**Statistic analysis**

Data are expressed as mean plus or minus the standard deviation (SD), and statistical analysis was performed by t-test. All the analyses were performed using GraphPad Prism 6. A value of *P* < 0.05 was accepted as significant.
